# Chlorophyllase, a Common Plant Hydrolase Enzyme with a Long History, Is Still a Puzzle

**DOI:** 10.3390/genes12121871

**Published:** 2021-11-24

**Authors:** Xueyun Hu, Imran Khan, Qingsong Jiao, Ahmad Zada, Ting Jia

**Affiliations:** 1 International Research Laboratory of Agriculture and Agri-Product Safety of the Ministry of Education of China, Yangzhou University, Yangzhou 225009, China; xyhulab@yzu.edu.cn (X.H.); jiaoqs@yzu.edu.cn (Q.J.); 2 Laboratory of Plant Functional Genomics of the Ministry of Education, Yangzhou University, Yangzhou 225009, China; 3 College of Bioscience and Biotechnology, Yangzhou University, Yangzhou 225009, China; dh18006@yzu.edu.cn (I.K.); dh19025@yzu.edu.cn (A.Z.)

**Keywords:** chlorophyllase, chlorophyll metabolism, localization

## Abstract

Chlorophyllase (Chlase, CLH) is one of the earliest discovered enzymes present in plants and green algae. It was long considered to be the first enzyme involved in chlorophyll (Chl) degradation, while strong evidence showed that it is not involved in Chl breakdown during leaf senescence. On the other hand, it is possible that CLH is involved in Chl breakdown during fruit ripening. Recently, it was discovered that *Arabidopsis* CLH1 is located in developing chloroplasts but not in mature chloroplasts, and it plays a role in protecting young leaves from long-term photodamage by catalysing Chl turnover in the photosystem II (PSII) repair cycle. However, there remain other important questions related to CLH. In this article, we briefly reviewed the research progress on CLH and listed the main unanswered questions related to CLH for further study.

Chlorophyllase (Chlase) is an enzyme that catalyses the conversion of chlorophyll (Chl) to chlorophyllide (Chlide) by removing the phytol side chain. It is one of the oldest enzymes found in plants [1]. Since it was discovered in 1912, Chlase has been extensively investigated. In the early stages, research on Chlase was mainly focused on its purification and properties from different plant species and algae [2,3]. Chlase activity existed in almost all of the tested plants, diatoms and green algae. It was suggested that Chlase is bound to the intracellular membrane, and its activity is latent, because hydrolysis of endogenous Chl only takes place when chloroplast membranes are disrupted or solubilized with detergents [4,5,6]. Both Chls (such as Chl *a*, Chl *b*, bacterioChl *a* and ProtoChl *a*, with magnesium) and pheophytins (Phetins) (such as Phetin *a* and PyroPhetin *a*, without magnesium) can be substrates of Chlase [7,8]. The reaction usually requires the presence of organic solvents, such as a high percentage of acetone or methanol solution [9]. For a long time, Chlase was considered the first enzyme for catalysing Chl degradation in senescent leaves [10,11,12]. One of the major reasons for this assumption is that its activity has been correlated with reduced Chl contents in senescing leaves and in the response to ethylene during fruit ripening [10,13]. However, Chlase activity is also present in green tissues and greening seedlings, suggesting a role in Chl turnover [8], as well as in nongreen tissues [14]. In addition, Chlase activity is present in the envelope of chloroplasts [15].

The identification of genes (*CLHs*) that encode proteins (CLHs) possessing Chlase activity was first reported in 1999 [16,17]. Thereafter, Chlase specifically referred to CLHs, and more *CLH* genes were identified from different species, including plants, green algae, and even cyanobacteria [18,19,20,21,22]. Indeed, although plants possess other enzymes that can catalyse the removal of the phytol side chain from Chl or Phetin [23,24,25], CLH has the highest activity against Chls. Surprisingly, not all CLHs contain predicted chloroplast transit peptides, such as *Ca*CLH (*Chenopodium album* CLH) and *At*CLH1 (*Arabidopsis* CLH1) [16]. Furthermore, *Ca*CLH contains a typical endoplasmic reticulum (ER) transit peptide, but does not possess the signal sequence for ER retention [16,26]. Therefore, *Ca*CLH might be imported to organelles other than the chloroplasts [27]. On the other hand, *Citrus* CLH and *At*CLH2 possess atypical transit peptides for chloroplasts [17]. At that stage, CLHs were still considered as the key enzymes for Chl degradation. Both inside and outside plastidial Chl degradation pathways related to CLHs were suggested.

The subcellular localization of CLH is one of the most important hints for uncovering the physiological function of it. In early times, CLH was repeatedly found in chloroplast fractions, and its activity was associated with chloroplast membranes [4,10,15,28,29,30,31,32] (Figure 1). Detailed investigation of the localization of CLHs began in 2007 [33]. Green fluorescent protein (GFP)-fused *At*CLH1 and *At*CLH2 were both observed outside of chloroplasts, which implies that neither *At*CLH isoform localizes to chloroplasts. In addition, it was found that the subcellular localization of *Chlamydomonas reinhardtii* CLH1-GFP was also outside the chloroplast [19]. By phenotype analysis, it was discovered that *clh1* and *clh2* single and double knockout lines are still able to degrade Chl during senescence. More detailed studies on the subcellular localization were performed later, and both native *At*CLH1 and *At*CLH2 were found to be located in the ER and tonoplast by organelle isolation and membrane fractionation, together with Western blotting [34,35]. In addition, *Arabidopsis* plants overexpressing *At*CLH1 and *At*CLH2 did not show a distinct phenotype from wild-type plants under normal growth conditions. Mis-targeting of *At*CLH1 to chloroplasts after estradiol induction causes the formation of Chlide and results in the cell death of the induced tissue upon illumination [34]. The results imply that locating *At*CLH1 in chloroplasts will damage the photosystem (PS) under light conditions. Furthermore, these mutant plants were able to degrade Chl at a similar rate as wild-type plants [33,34,35], and there was no visible Chl metabolism-related phenotype. If CLHs are involved in Chl degradation, their knockout mutant plants should show a stay-green phenotype during dark-induced leaf senescence, as inferred from the phenotype of plants lacking one of the other Chl degradation enzymes [23,36]. Moreover, another pathway for Chl degradation involves pheophytinase (PPH), another enzyme that catalyses the dephytylation of Phetin *a*, which was demonstrated to be the essential enzyme for catalysing Chl degradation in *Arabidopsis* [23]. Taken together, *At*CLHs are not involved in Chl degradation during leaf senescence. On the other hand, it was reported that most *Citrus* CLHs are located in chloroplasts [37], and neither N- nor C-terminal-processed domains are essential for chloroplast targeting of this enzyme [38]. In addition, *Citrus* CLH responds to ethylene treatment, and Chl levels were negatively correlated with plastid CLH accumulation. Therefore, *Citrus* CLH is suggested to play a central role in Chl breakdown during *Citrus* fruit colour break. Considering that the amino acid sequences of CLHs are not very conserved across species and PPH is the core dephytylation enzyme during leaf senescence, but not during fruit ripening in tomato, it is possible that the function of CLHs varies in species or plant organs.

Most recently, a report revealed additional information on CLHs [39,40]. It was found that *Arabidopsis At*CLH1 was localized to the developing chloroplasts of young leaves but was located in the cytosol of mature leaves [39]. Leaves from CLH1-null mutant seedlings and those from CLH1- and CLH2-overexpression seedlings were ~25% lower and ~30% higher Chl contents, respectively. It is a pity that these Chl-related phenotypes were not observed in previous studies [33,34,35]. Different growth conditions may be responsible for the different phenotypes of *clh* mutants, and also CLH overexpression lines described in the above literature. Consistent with *Citrus* CLH, the N-terminus of *At*CLH1 is also not essential for its chloroplast localization. In developing chloroplasts, *At*CLH1 associates with the PSII-dismantling complex PSII core monomer (RCC1) and CP43-less PSII core monomer (RC47). The transcript and protein levels of *At*CLH1 are upregulated, and *At*CLH1 binds primarily to RC47 upon exposure to high light. Seedlings of *clh1* single and *clh1-1/2-2* double mutants display increased high-light sensitivity, whereas seedlings overexpressing *At*CLH1 have enhanced high-light tolerance compared with the wild-type. Furthermore, considering that *At*CLH1 interacts with the AAA domain of thylakoid protease FtsH2, and *At*CLH1 overexpression suppress the variegation of *var2-2* mutants that lack FtsH2 and restore D1 degradation. It was suggested that *At*CLH1 primarily catalyzes the dephytylation of Chl *a* molecules bound by the photodamaged D1 protein in RC47; thus, the released apo-D1 protein can be unfolded and proteolyzed by FtsH complexes. Therefore, *At*CLH1 is involved in PSII repair and function in the long-term adaptation of young leaves to high light exposure by facilitating FtsH-mediated D1 degradation.

Although there is a long research history on the underlying characteristics and function of CLH, much is still unknown about CLH.

1. Why are there different phenotypes when *At*CLH1 is overaccumulated in chloroplasts? Mis-targeting *At*CLH1 into chloroplasts caused serious cell death in *Arabidopsis* [29]. This result is reasonable because *At*CLH1 activity is very high when catalysing Chl dephytylation [16,24]. In another report, it was observed that inducing overexpression of STAY-GREEN (SGR), the enzyme that catalyses the extraction of Mg from Chl, caused fast Chl degradation and cell death in *Arabidopsis* too [41]. Moreover, it was reported that overexpression of the mature version of *Citrus* CLH in squash caused light-dependent lesion-mimic phenotypes, while overexpression of full-length CLH has no obvious phenotype [42]. Therefore, we infer that if CLH overaccumulates in chloroplasts and associates with photosynthetic proteins or complexes, Chl can be easily degraded by CLH. In that case, a PS cannot be established if plants continue overexpressing CLH in chloroplasts. However, Tian and coworkers showed that *At*CLH1 was overaccumulated in developing chloroplasts in *At*CLH1-overexpressing plants. The *At*CLH1-overexpressing plants showed a slightly darker green colour, with ~30% higher Chl contents under low light conditions. There are two possibilities to explain these different results. One is that the mistargeted *At*CLH1 and overexpressed *At*CLH1 have different sub-localizations inside the chloroplasts; thus, overexpressed *At*CLH1 cannot access Chl under low light conditions. Another possibility is that overexpressed *At*CLH1 has low activity in developing chloroplasts when plants are grown under low light conditions. These possibilities need further supporting evidence.
2. How can CLH be imported into developing chloroplasts but not imported into mature chloroplasts? It was reported that the plastid protein import apparatus was regulated by the developmental stage, and this regulation was signal peptide-dependent [43,44,45]. However, the N-terminus of *At*CLH1 is not essential for its chloroplast localization [39]. Therefore, further study is required to determine which sequence of *At*CLH1 is essential for its chloroplast targeting. Furthermore, whether *At*CLH1 can be imported into chloroplasts through the secretory pathway needs further investigation [46]. This possibility is supported by the fact that CLHs from different species are modified by glycosylation [16,47], an important modification for protein import into chloroplasts through the secretory pathway [46]. If this possibility is true, the secretory pathway for protein import into chloroplasts should also be age dependent.
3. What is the function of CLH that is located outside of chloroplasts? It was demonstrated that *At*CLHs are located in the ER and tonoplasts [34,35]. If CLH only functions in developing chloroplasts, why is it biosynthesized, and is its expression induced by MeJA if it cannot be imported into mature chloroplasts? It is possible that CLH also has a physiological function when it is located in the ER and/or tonoplast. It has been reported that CLH plays roles in disease, fungicide or insect defence, especially CLH and Chl, which form binary defence systems against chewing herbivores [34,48,49]. However, other possibilities also exist. First, CLH may catalyse Chl dephytylation in the extraplastidic Chl degradation pathway [27], although stress-induced cytosolic DUF538 proteins were suggested to be Chl hydrolyzing enzymes too [50]. Our previous studies showed that *At*CLHs are located in the tonoplast and ER membrane, which may fuse together with the Chl-containing vesicles that are derived from the chloroplast envelope under stress conditions [51,52,53]. In that case, CLHs may be able to target Chl and catalyse its dephytylation. Second, other substrates may be catalysed by extraplastidic CLH.
4. Does plastidic CLH possess different roles in Chl metabolism in fruits and leaves? Based on previous studies, *Citrus* CLH is located in chloroplasts, responds to ethylene and is involved in Chl degradation during fruit ripening [37], while plastidic *At*CLH1 functions in protecting young leaves from long-term photodamage by regulating PSII repair [39]. In addition, PPH is the core phytol-hydrolytic enzyme during leaf senescence; however, tomato (*Solanum lycopersicum*) fruit ripening involves other hydrolases [54]. If CLH is located inside of chloroplasts, it is possible that CLH is the enzyme involved in Chl degradation during tomato fruit ripening, similar to *Citrus* CLH. Thus, it is necessary to demonstrate whether *Sl*CLH is located in both the developing and mature chloroplasts of tomato fruit.
5. In addition to catalysing Chl dephytylation, CLH is shown to dephytylate Phetins in vitro [55]. Phetin is an important component of the reaction centre of PSII, if Phetin undergoes de- and rephytylation during PSII repair, it is interesting to investigate whether CLH is involved in catalysing Phetin dephytylation during PSII repair.

Taken together, although it was discovered more than 100 years ago and many studies have focused on its function and the related mechanisms, there are still many unanswered questions about CLH. Further studies on CLH will help us to know more about CLH, and also to uncover the mechanism of protein import into chloroplasts.

## Figures and Tables

**Figure 1:**
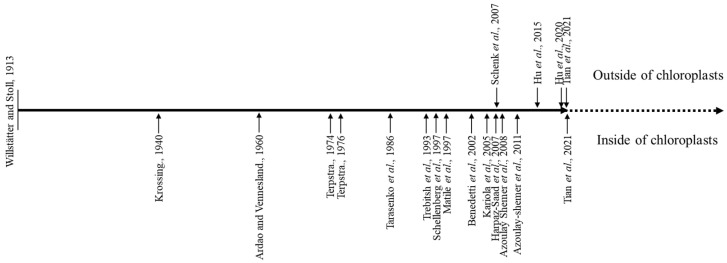
Research progress on the subcellular localization of Chlase. Above lists the publications that showed Chlase located outside of chloroplasts; below lists the studies that reported Chlase located inside of chloroplasts.

## Data Availability

Not applicable.
